# Relationship between Smoking and Obesity: A Cross-Sectional Study of 499,504 Middle-Aged Adults in the UK General Population

**DOI:** 10.1371/journal.pone.0123579

**Published:** 2015-04-17

**Authors:** Shadrach Dare, Daniel F. Mackay, Jill P. Pell

**Affiliations:** 1 Institute of Health and Wellbeing, University of Glasgow, Glasgow, United Kingdom; 2 School of Nursing, College of Health and Allied Sciences, University of Cape Coast, Cape Coast, Ghana; Kyushu University Faculty of Medical Science, JAPAN

## Abstract

**Background:**

There is a general perception that smoking protects against weight gain and this may influence commencement and continuation of smoking, especially among young women.

**Methods:**

A cross-sectional study was conducted using baseline data from UK Biobank. Logistic regression analyses were used to explore the association between smoking and obesity; defined as body mass index (BMI) >30kg/m^2^. Smoking was examined in terms of smoking status, amount smoked, duration of smoking and time since quitting and we adjusted for the potential confounding effects of age, sex, socioeconomic deprivation, physical activity, alcohol consumption, hypertension and diabetes.

**Results:**

The study comprised 499,504 adults aged 31 to 69 years. Overall, current smokers were less likely to be obese than never smokers (adjusted OR 0.83 95% CI 0.81-0.86). However, there was no significant association in the youngest sub-group (≤40 years). Former smokers were more likely to be obese than both current smokers (adjusted OR 1.33 95% CI 1.30-1.37) and never smokers (adjusted OR 1.14 95% CI 1.12-1.15). Among smokers, the risk of obesity increased with the amount smoked and former heavy smokers were more likely to be obese than former light smokers (adjusted OR 1.60, 95% 1.56-1.64, p<0.001). Risk of obesity fell with time from quitting. After 30 years, former smokers still had higher risk of obesity than current smokers but the same risk as never smokers.

**Conclusion:**

Beliefs that smoking protects against obesity may be over-simplistic; especially among younger and heavier smokers. Quitting smoking may be associated with temporary weight gain. Therefore, smoking cessation interventions should include weight management support.

## Introduction

Smoking and obesity are major public health challenges and the prevalence of both is increasing globally. Smoking increases the risk of cancer, respiratory and cardiovascular diseases, and is the leading preventable cause of death in developed countries [1]. Obesity is the fifth leading cause of death, globally, and accounts for 44% of cases of diabetes and 23% of ischaemic heart disease [2 3]. The Framingham Study showed that the life expectancy of obese smokers is around 13 years shorter than non-obese, never smokers [4].

Over 80% of smokers wish to quit smoking but only 33% attempt to do so [5 6]. Of those who attempt to quit, 75%-80% relapse within six months [7]. Addiction is the main reason for smokers failing to quit. However, concerns about weight gain are an independent factor in smokers deciding not to quit, especially young women [8]. Also, the general perception that smoking may protect against obesity is a common reason for starting smoking among adolescents [9].

The relationship between smoking and obesity is complex and not completely understood, and published studies have produced conflicting results. While some studies have shown no significant association between smoking status and body mass index (BMI) [10], others have suggested that smoking may be associated with lower BMI [11] and smoking cessation with increased BMI [12].

It is possible that the association reflects reverse causation due to overweight individual, who are trying to lose weight, being more likely to start smoking [13]. However, previous studies have also explored possible causal mechanisms. The most robust evidence, to date, supports a peripheral metabolic effect. Administration of nicotine to animal models has been associated with reduced weight, in the absence of reduced calorific intake, due to less efficient absorption and storage of calories and increased metabolic rate and thermogenesis [13–15]. Since nicotine is a cholinergic agonist and readily crosses the blood brain barrier, a central effect on eating is hypothetically plausible but yet to be established. Similarly, the evidence for reduced calorific intake due to smoking being a replacement behaviour, impairing smell or taste, or changing food preference is largely anecdotal.

Previous studies have tended to focus on overall associations and there is a general paucity of studies examining whether the associations are moderated by age, gender or socioeconomic status. Therefore, the aim of this study was to explore the overall relationship between smoking and obesity in the UK general population; to determine whether it varies between sub-groups of the population; and whether there was evidence of a dose-relationship.

## Methods

We conducted a cross-sectional study using baseline data from the UK Biobank cohort study. Between the years 2006 and 2010, UK Biobank recruited 502,682 members of the general public, aged 31 to 69 years, via 22 research clinics located across the United Kingdom, and conducted a detailed baseline survey. Information collected from these middle-aged adults included socio demographic characteristics, physical activity measurements, past medical and surgical history, lifestyle risk factors (including smoking and alcohol consumption), anthropometric measures (including height and weight) and biological samples. Details of the protocol have been published earlier [16 17]. BMI was derived from weight and height: weight (kg) / (height (m) x height (m)). Participants removed their shoes and heavy outer clothing before weight and height were measured. Weight was measured, to the nearest 0.1kg, using the Tanita BC-418 MA body composition analyser. Height was measured using a Seca 202 height measure. Obesity was defined as a BMI of >30 kg/m^2^.

Smoking behaviour was recorded via a self-completed, touch-screen questionnaire. The information collected included: current smoking status, amount smoked, duration of smoking and time since quitting smoking. Smoking status was categorised into: current, former or never smoker. The amount smoked by current smokers was assessed in three ways: number of cigarettes smoked (per day), duration of smoking (in years), and lifetime consumption of cigarettes (pack years). Participants provided information on the first two and pack years was derived from: (number of cigarettes smoked (per day) x duration of smoking (in years)) / 20. The number of cigarettes smoked per day was used to categorise current smokers into: heavy (>20 cigarettes per day), moderate (10–20 cigarettes per day) and light (<10 cigarettes per day) smokers.

The amount previously smoked by former smokers was assessed in two ways: amount smoked and duration of smoking (in years). In relation to the amount smoked, former smokers were asked whether they had smoked on most/all days or were occasional smokers. The latter were then asked whether they had smoked at least 100 cigarettes over their lifetime or not. These two variables were combined to classify former smokers into: heavy (smoked most/all days), moderate (occasional smoker who smoked at least 100 cigarettes in total) and light (occasional smoked who smoked less than 100 cigarettes in total) former smokers. Both former and current smokers were included in the analyses of time (in years) since quitting with time recorded as 0 for current smokers.

Physical activity and alcohol consumption were self-reported. Physical activity was self-reported number as the number of days per week on which participants walked for at least 10 minutes. Alcohol intake was also self-reported as the frequency with which they consumed alcohol: never, occasionally, 1–3 times per month, 1–2 times per week, 3–4 per week and daily. Hypertension and diabetes were defined as self-report of a doctor diagnosis. Socioeconomic deprivation was derived from the Townsend (1988) Index which is an area-based measure of material deprivation [18]. Census data on the percentage of households without a car, overcrowded, not owner-occupied and containing unemployed residents were converted into z scores which were summated to provide a Townsend score. This was used to derive deciles for the study population.

Participants were excluded from the analyses if data on height or weight were missing. The characteristics of obese and non-obese participants were compared using chi square tests for binary variables and chi square tests for trend for ordinal variables. Binary logistic regression analyses were undertaken using obesity (yes/no) as the outcome. Multivariate analyses were undertaken to adjust for the potential confounding effects of: age, sex, socioeconomic deprivation decile, physical activity, alcohol consumption, hypertension and diabetes. The Hosmer-Lameshow test was used to assess the adequacy of the models. We formally tested for interactions with age, gender and socioeconomic deprivation decile, and undertook sub-groups analyses where the results were statistically significant. All analyses were undertaken using Stata version 12.0.

Permissions to conduct UK Biobank were received from the North West Multi-centre Research Ethics Committee, the England and Wales Patient Information Advisory Group, and the Scottish Community Health Index Advisory Group. Individual informed, written consent was obtained from participants prior to data collection. All data users are required to sign a written agreement with UK Biobank and the data extract is anonymised.

## Results

Of the 502,682 UK Biobank participants, 3,178 (0.6%) were excluded due to missing data on height, weight or both. The remaining 499,504 participants comprised the study population. Of these, 122,284 (24.5%) were obese, 38,875 (7.8%) were current smokers, 258,872 (52.0%) were former smokers and 199,814 (40.2%) were never smokers. The prevalence of obesity was higher among participants who were male, aged over 50 years, had hypertension or diabetes (Table 1). It increased with increasing socioeconomic deprivation, decreasing physical activity and decreasing alcohol consumption (Table 1).

**Table 1:** Characteristics of obese and not obese participants.

	Not Obese (N = 377,220)	Obese (N = 122,284)	P value*
	N (%)	N (%)	
**Age (years)**			
31–40	31,493 (8.3)	8,508 (7.0)	<0.001
41–50	105,116 (27.9)	32,691 (26.7)	
51–60	152,997 (40.6)	52,308 (42.8)	
≥61	87,614 (23.2)	28,777 (23.5)	
**Gender**			
Female	207,664 (55.1)	64,326 (52.6)	<0.001
Male	169,556 (44.9)	57,958 (47.4)	
**Deprivation decile**			
1 (most affluent)	40,044 (10.6)	10,018 (8.2)	<0.001
2	39,915 (10.6)	10,376 (8.5)	
3	39,019 (10.4)	10,729 (8.8)	
4	38,765 (10.3)	11,228 (9.2)	
5	38,507 (10.2)	11,449 (9.4)	
6	37,853 (10.0)	12,041 (9.9)	
7	37,459 (9.9)	12,432 (10.2)	
8	36,644 (9.7)	13,213 (10.8)	
9	35,217 (9.3)	14,481 (11.9)	
10 (most deprived)	33,353 (8.9)	16,149 (13.2)	
Missing	444	168	
**Number of days per week participant walks >10 minutes**			
0	6,293 (1.7)	3,979 (3.4)	<0.001
1	9,370 (2.5)	4,006 (3.4)	
2	21,134 (5.7)	8,728 (7.5)	
3	28,338 (7.6)	10,868 (9.3)	
4	29,685 (8.0)	10,190 (8.8)	
5	59,056 (15.9)	20,647 (17.7)	
6	38,202 (10.3)	11,680 (10.0)	
7	179,406 (48.3)	48,311 (41.5)	
Missing	5,736	3,875	
**Alcohol consumption**			
Never	27,556 (7.3)	12,552 (10.3)	<0.001
Occasionally	38,371 (10.2)	19,219 (15.8)	
1–3 per month	39,228 (10.4)	16,382 (13.4)	
1–2 per week	96,831 (25.7)	31,919 (26.2)	
3–4 per week	91,836 (24.4)	23,173 (19.0)	
Daily	82,646 (22.0)	18,700 (15.3)	
Missing	752	339	
**Hypertension**			
No	293,635 (78.1)	69,271 (56.9)	<0.001
Yes	82,356 (21.9)	52,481 (43.1)	
Missing	1,229	532	
**Diabetes**			
No	363,790 (96.8)	107,486 (88.5)	<0.001
Yes	12,090 (3.2)	13,997 (11.5)	
Missing	1,340	801	

N number.

*chi square test for gender, hypertension and diabetes, chi-square test for trend for remainder.

### Smoking status

Obesity was most prevalent among former smokers and least prevalent among current smokers (Table 2). Univariate analyses confirmed that current smokers were less likely to be obese than never smokers (OR 0.91, 95% CI 0.89–0.93, p<0.001), and former smokers were more likely to be obese than both current smokers (OR 1.20, 95% CI 1.17–1.23, p<0.001) and never smokers (OR 1.09, 95% CI 1.08–1.11, p<0.001). The results remained statistically significant after adjusting for age, gender, socioeconomic deprivation decile, levels of physical activity and alcohol consumption, and the presence of hypertension and diabetes. The reduced risk among current smokers was slightly attenuated (adjusted OR 0.83, 95% CI 0.81–0.86, p<0.001), and the increased risk among former smokers was increased in comparison with both current smokers (adjusted OR 1.33, 95% CI 1.30–1.37, p<0.001) and never smokers (adjusted OR 1.14, 95% CI 1.12–1.15, p<0.001). The Hosmer-Lameshow test produced a p value of 0.32, indicating that the model was an adequate fit.

**Table 2:** Smoking behaviour of obese and not obese participants.

	Not Obese	Obese	P value*
	(N = 377,220)	(N = 122,284)	
	N (%)	N (%)	
**Smoking status**			
Current	30,301 (8.1)	8,574 (7.0)	<0.001
Former	193,182 (51.4)	65,690 (54.0)	
Never	152,413 (40.5)	47,401 (39.0)	
Missing	1,324	619	
**Current smokers**			
**Number of cigarettes (per day)**			
<10	5,950 (21.3)	1,201 (15.3)	<0.001
10–20	18,369 (65.6)	5,130 (65.5)	
>20	3,662 (13.1)	1,499 (19.1)	
Missing	2,320	744	
**Duration of smoking (years)**			
<10	529 (1.8)	163 (1.9)	0.327
11–20	1,653 (5.5)	504 (6.0)	
21–30	8,210 (27.5)	2,202 (26.1)	
≥31	19,460 (65.2)	5,579 (66.0)	
Missing	449	126	
**Pack years**			
<10	4,338 (16.4)	919 (12.4)	<0.001
11–20	6,541 (24.8)	1,666 (22.5)	
21–30	6,470 (24.5)	1,738 (23.5)	
31–40	4,547 (17.2)	1,379 (18.6)	
41–50	2,551 (9.7)	904 (12.2)	
≥51	1,964 (7.4)	803 (10.8)	
Missing	3,890	1,165	
**Former smokers**			
**Amount formerly smoked**			
Occasional smoker, <100 cigarettes in total	56,775 (30.3)	14,239 (22.2)	<0.001
Occasional smoker, ≥100 cigarettes in total	46,477 (24.8)	13,721 (21.4)	
Smoked most/all days	84,293 (44.9)	36,110 (56.4)	
Missing	5,637	1,620	
**Duration of former smoking (years)**			
<10	16,164 (19.4)	4,634 (13.0)	<0.001
11–20	26,290 (31.5)	9,900 (27.7)	
21–30	20,357 (24.4)	10,160 (28.4)	
≥31	20,591 (24.7)	11,069(31.0)	
Missing	104,143	28,307	
**Former and current smokers**			
**Time since quitting (years)**			
0	30,301 (29.2)	8,574 (21.5)	<0.001
1–10	20,784 (20.0)	10,476 (26.2)	
11–20	19,517 (18.8)	9,117 (22.8)	
21–30	21,832 (21.0)	8,369 (20.9)	
31	11,361 (10.9)	3,420 (8.6)	

N number.

*chi-square test for trend for all variables.

Statistically significant interactions were found between smoking status and age, gender and socioeconomic deprivation decile (all p<0.001). The reduced risk of obesity among current smokers was less pronounced in men and younger participants and did not reach statistical significance among participants of 40 years of age or younger (Table 3). There was a trend across socioeconomic deprivation deciles. Among the most deprived 40% of participants, current smokers had a significantly reduced risk of obesity. However, there was no significant association in more affluent sub-groups and, in the most affluent 20% there was a non-significant increased risk of obesity among current smokers.

**Table 3:** Multivariate* binary logistic regression analysis of the association between smoking status and obesity stratified by gender, age and socioeconomic deprivation decile.

	Never smokers	Current smokers	P value	Former smokers	P value
		OR (95% CI)		OR (95% CI)	
**Gender**					
Female	1.00	0.83 (0.79–0.86)	<0.001	1.09 (1.07–1.12)	<0.001
Male	1.00	0.86 (0.83–0.90)	<0.001	1.21 (1.18–1.24)	<0.001
**Age (years)**					
31–40	1.00	0.92 (0.84–1.00)	0.064	0.99 (0.93–1.04)	0.604
41–50	1.00	0.84 (0.80–0.88)	<0.001	1.05 (1.02–1.08)	0.001
51–60	1.00	0.82 (0.78–0.86)	<0.001	1.16 (1.14–1.19)	<0.001
≥61	1.00	0.76 (0.71–0.82)	<0.001	1.28 (1.24–1.32)	<0.001
**Socioeconomic deprivation decile**					
1 (most affluent)	1.00	1.11 (0.99–1.26)	0.078	1.20 (1.14–1.26)	<0.001
2	1.00	1.03 (0.91–1.15)	0.676	1.23 (1.17–1.29)	<0.001
3	1.00	0.95 (0.85–1.07)	0.403	1.23 (1.17–1.29)	<0.001
4	1.00	0.87 (0.78–0.98)	0.016	1.15 (1.10–1.21)	<0.001
5	1.00	0.97 (0.88–1.08)	0.583	1.19 (1.13–1.24)	<0.001
6	1.00	0.93 (0.85–1.02)	0.146	1.13 (1.08–1.18)	<0.001
7	1.00	0.87 (0.79–0.95)	0.002	1.18 (1.12–1.23)	<0.001
8	1.00	0.84 (0.77–0.91)	<0.001	1.10 (1.05–1.15)	<0.001
9	1.00	0.76 (0.71–0.82)	<0.001	1.05 (1.01–1.10)	0.022
10 (most deprived)	1.00	0.62 (0.59–0.66)	<0.001	0.95 (0.91–0.99)	0.026

OR odds ratio, CI confidence interval.

*adjusted for levels of physical activity and alcohol consumption, and presence of hypertension and diabetes as well as gender, age, and socioeconomic deprivation decile as appropriate.

The increased risk of obesity among former smokers was also less pronounced in women and not statistically significant in the youngest group of participants (Table 3). The trend across socioeconomic deciles was in the opposite direction to current smokers. The association between former smokers and increased risk of obesity was strongest in the most affluent groups and fell with increasing deprivation. In the most deprived 10% of the study population, former smokers had a significantly reduced risk of obesity in comparison with never smokers.

### Current smokers

**Fig 1:**
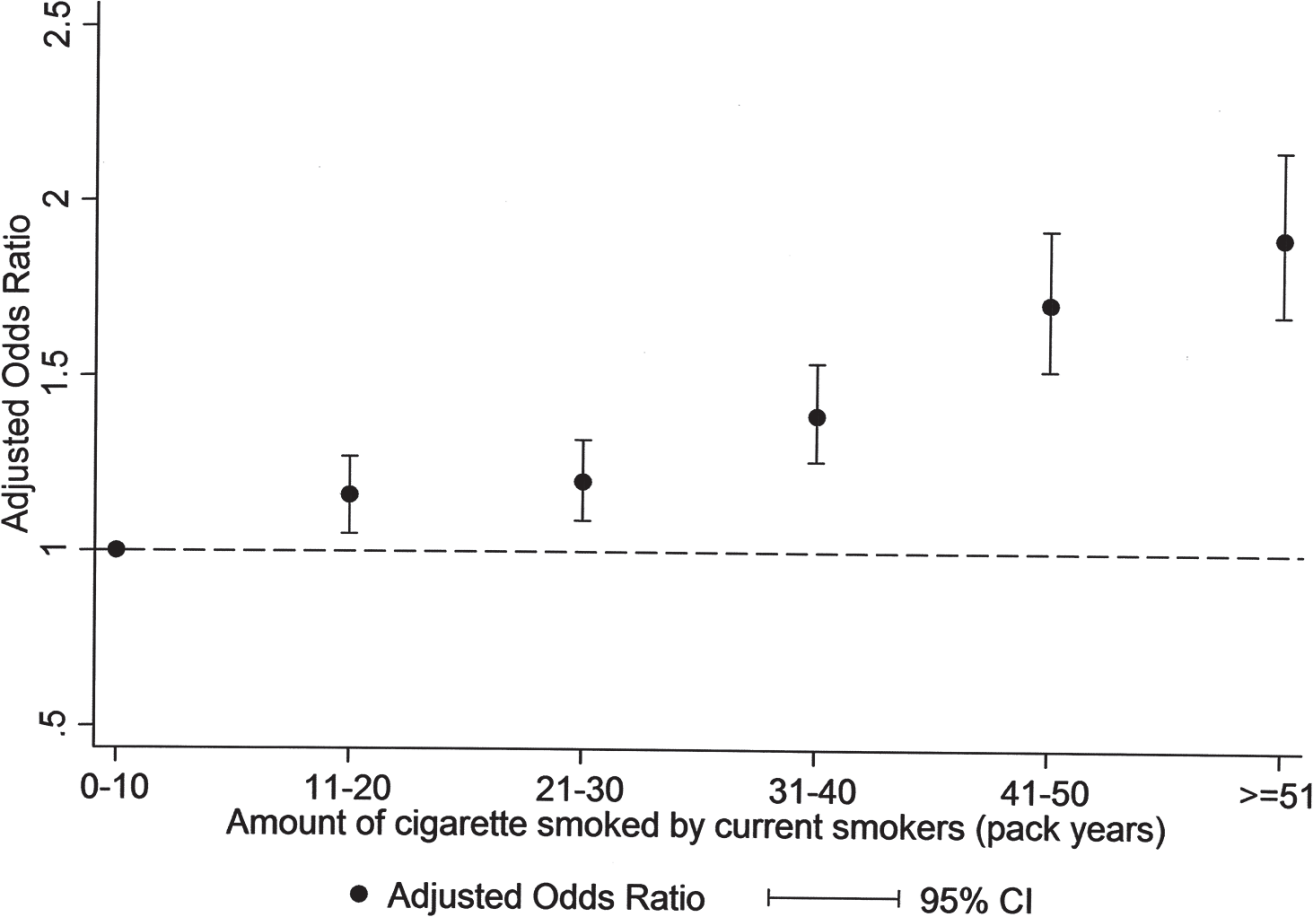
Forest plot of adjusted* odds ratio for obesity and lifetime consumption of cigarette smoked (pack years) among current smokers. * adjusted for levels of physical activity and alcohol consumption, and presence of hypertension and diabetes as well as gender, age, and socioeconomic deprivation decile.

Among current smokers, the prevalence of obesity did not vary significantly according to duration of smoking but did increase with increasing number of cigarettes smoked per day and, therefore, also with pack years (Table 2). Logistic regression analysis showed a dose relationship between the number of cigarettes smoked daily by current smokers and obesity. Univariate analyses showed that current heavy smokers (>20 cigarettes per day) were more likely to be obese than both moderate (10–20 cigarettes per day) (OR 1.47, 95% CI 1.37–1.57, p<0.001) and light (<10 cigarettes per day) smokers (OR 2.03, 95% CI 1.86–2.21, p<0.001), and moderate smokers were more likely to be obese than light smokers (OR 1.38, 95% CI 1.29–1.48, p<0.001). The results remained statistically significant after adjusting for age, gender, socioeconomic deprivation decile, level of physical activity and alcohol consumption, and the presence of hypertension and diabetes. However, compared with light smokers, the increased risk was slightly attenuated among both heavy (adjusted OR 1.86, 95% CI 1.70–2.05, p<0.001) and moderate (adjusted OR 1.28, 95% CI 1.39–1.37, p<0.001) smokers. Among current smokers, there was also a dose relationship between pack years and obesity (Fig 1). Compared to current smokers with less than 10 pack years of consumption, the adjusted odds of obesity for those with >50 pack years was 1.90 (95% CI 1.68–2.15, p<0.001). In comparison with never smokers, light (adjusted OR 0.65, 95% CI 0.61–0.70, p<0.001) and moderate (adjusted OR 0.80, 95% CI 0.77–0.82, p<0.001) current smokers were less likely to be obese. However, heavy smokers were more likely to be obese than never smokers (adjusted OR 1.09, 95% CI 1.02–1.17, p = 0.01).

### Former smokers

Among former smokers, the prevalence of obesity increased with both the amount previously smoked and the previous duration of smokers, and the prevalence was highest among those who had quit within the last ten years and lowest among those who had quit more than thirty years previously (Table 2). In the multivariate analyses, there was still a dose-relationship with the amount smoked by former smokers. In comparison to former light smokers, obesity was more likely among both former moderate smokers (adjusted OR 1.20, 95% CI 1.16–1.23, p<0.001) and former heavy smokers (adjusted OR 1.60, 95% CI 1.56–1.64, p<0.001). In comparison with never smokers, former heavy (adjusted OR 1.40, 95% CI 1.37–1.42, p<0.001) and moderate (adjusted OR 1.04, 95% CI 1.02–1.06, p = 0.001) smokers were more likely to be obese, but former light smokers (adjusted OR 0.87, 95% CI 0.85–0.89, p<0.001) were less likely to be obese. On multivariate analysis, risk of obesity increased with the duration of previous smoking (years) and then plateaued. In comparison with former smokers who had previously smoked for 10 years or less, the increased risk of obesity was identical among those who had smoked for 21–30 years and >30years (both: adjusted OR 1.60, 95% CI 1.56–1.64, p<0.001).

**Fig 2:**
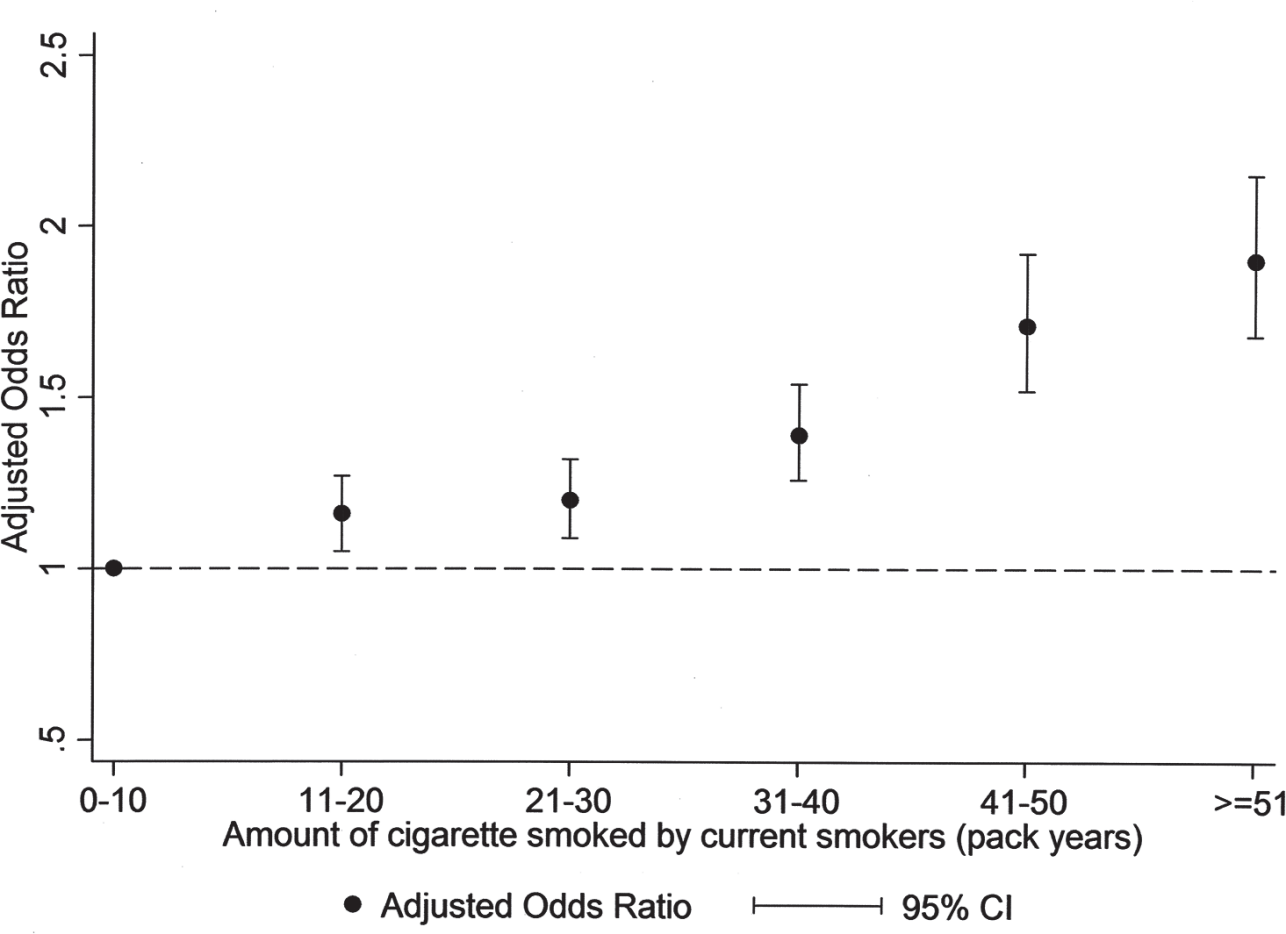
Forest plot of adjusted* odds ratio for obesity and duration since quitting smoking among former smokers. * adjusted for levels of physical activity and alcohol consumption, and presence of hypertension and diabetes as well as gender, age, and socioeconomic deprivation decile.

Fig 2 shows the results of the multivariate analyses examining the effect of time since quitting on the risk of obesity among former smokers. In Fig 2A, former smokers are compared to current smokers and in Fig 2B to never smokers. In both the increased risk of obesity in former smokers falls over. Smokers who quit more than 30 years previously still had significantly higher risk of obesity than current smokers (adjusted OR 1.11, 95% CI 1.05–1.17. p<0.001) but were not significantly different from never smokers (adjusted OR 1.03, 95% CI 0.98–1.07, p = 0.22).

## Discussion

Overall associations between smoking and obesity masked important sub-group differences. Overall, current smokers were less likely to be obese than never smokers but this was not true among younger participants and those living in the most affluent areas of residence. Also, the association was reversed among heavy smokers who were more likely to be obese than never smokers. Similarly, former smokers were more likely to be obese overall, but this was not true of former light smokers, was less pronounced in women and was not statistically significant in the youngest age-group and those who had quit a long time previously. The increased risk of obesity among former smokers decreased with increasing deprivation and was reversed in the most deprived 10% of participants.

Both smokers and non-smokers believe that smoking is an effective way of reducing body weight [9]. Concerns about weight exert significant influence over decisions to commence and quit smoking among young people, particularly women [9 19], and fear of weight gain has been cited as a cause of relapse among former smokers [8]. Both obesity [20 21] and smoking [22] are more common among socioeconomically deprived individuals.

Our finding of lower adiposity among current smokers, overall, is consistent with a number of previous cross-sectional [23 24] and cohort [25–31] studies, although the opposite has been reported by some studies [32]. Similarly, our finding of increased obesity among former smokers corroborates studies by Reas et al [33] who reported increased BMI following smoking cessation and Basterra-Gortari et al [32] who reported higher BMI in former than never smokers. The former study reported higher absolute weight gains in women than men, while the latter reported the opposite. There is a paucity of studies examining whether age and deprivation moderate the association between smoking and adiposity, in relation to both current and former smoking. In our study, there was no evidence that smoking protected against obesity among younger and deprived people.

Heavy smokers may be more concerned about their weight than light smokers [19]. However, our study demonstrated increasing risk of obesity with increasing consumption of cigarettes, whether measured by number of cigarettes smoked daily or pack years. Several previous studies have found similar associations with the number of cigarettes smoked daily [23 25 34 35]. Stavropoulos-Kalinoglou et al [36] reported a negative correlation between pack-years and total body fat. However, their study was conducted on 392 patients with rheumatoid arthritis, a condition which causes muscle wasting [37 38]. They demonstrated a significantly lower fat free mass among heavy smokers and their results may not be generalizable to the general population. In a longitudinal study of 7,565 participants, Basterra-Gortari et al., demonstrated greater weight gain among active than never smokers over a 50 month follow-up period [32].

Previous studies have tended to dichotomize former smokers into recent and longer term quitters and shown lower adiposity in the latter [33 39]. We were able to demonstrate a dose relationship with increasing time from quitting, with the risk of obesity falling to a level not significantly different from never smokers more than 30 years after quitting. However, in comparison to current smokers, former smokers were still at increased risk of obesity even more than 30 years after quitting. This is contrary to a study by Raes et al [33] that demonstrated no significant difference in weight between current smokers and those who had quit more than five years previously. However, this was based on BMI derived from self-reported weight and height. Self-reported height tends to be overestimated and weight underestimated, particularly by women [40 41], leading to underestimation of body mass index.

Since randomization to smoking would be unethical, studies on this topic are inevitably observational. This was a cross-sectional study. Therefore, it is not possible to establish temporal relationship and reverse causation is possible. UK Biobank is representative of the UK general population in the relevant age-band in terms of age, gender, socioeconomic status and ethnicity. In common with other cohort studies, it is unrepresentative in terms of health-related behaviours. There is a lower prevalence of adverse lifestyle factors, such as smoking. Therefore, care should be taken in generalizing absolute values and differences, as opposed to relative differences. It is also important to check whether overall associations are moderated by demographic characteristics. Due to the large size of this study, we were able to test for interactions, undertake sub-group analyses and explore dose relationships, as well as examining overall associations.

UK Biobank collected a wide range of variables, enabling us to adjust for potential confounders. Whilst every effort was made to adjust for potential confounding factors, residual confounding is always possible. Information on smoking status, the amount smoked and duration were all self-reported. There was no corroboration nor validation via biochemical assays. However, there is good agreement between self-reported smoking status and cotinine concentrations in studies conducted on the general population [42]. Furthermore, it is unlikely that any error in smoking data would be systematically related to the presence of obesity. Data on body mass index were measured, rather than self-reported. Categorisation of BMI into obese and non-obese was consistent with previous studies and with the tendency to target both clinical and public health interventions on the basis of cut-offs. However, the reality is that the increasing risk of adverse health outcomes associated with increasing BMI is a gradient across the whole spectrum of BMI above normal weight. Therefore, categorization inevitably loses some information on the overall association.

This study corroborated the perception that, overall, current smokers are less likely to be obese than never smokers and former smokers are more likely to be obese than current smokers. However, association does not necessarily imply causation. There is a lack of consensus on whether an association between smoking status and obesity could be causal. Possible causal mechanisms include reduced calorific intake, due to a central effect, impaired smell or taste, a change in food preference, or a direct metabolic effect on the absorption or storage of calories, or increased energy expenditure [43–45]. However, reverse causation may be an alternative explanation. Evidence supportive of causation includes reversibility and a dose relationship. The fact that cessation has the opposite effect from commencement supports reversibility. However, there was a negative dose relationship whereby heavier smokers were at significantly increased risk of obesity, rather than reduced risk. This argues against causation. Also, the overall association masks important sub-group differences. Young people and women are more likely to be influenced by weight concerns when deciding whether to commence or quit smoking. However, the evidence for any protective effect of smoking against weight gain is much weaker in these sub-groups.
