# Correction: Relationship between Smoking and Obesity: A Cross-Sectional Study of 499,504 Middle-Aged Adults in the UK General Population

**DOI:** 10.1371/journal.pone.0172076

**Published:** 2017-02-08

**Authors:** Shadrach Dare, Daniel F. Mackay, Jill P. Pell

The image for Fig 2 is a duplicate of Fig 1. Please see the complete, corrected Fig 2 here.

**Fig 2:**
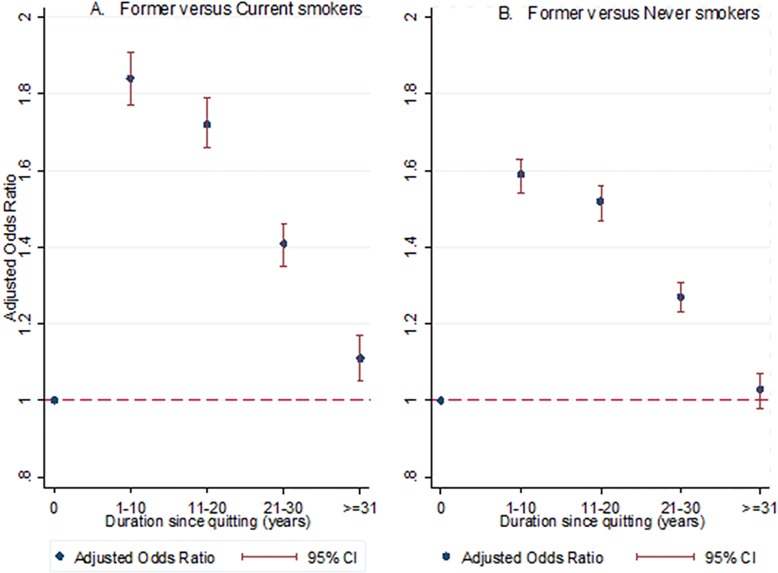
Forest plot of adjusted* odds ratio for obesity and duration since quitting smoking among former smokers. * adjusted for levels of physical activity and alcohol consumption, and presence of hypertension and diabetes as well as gender, age, and socioeconomic deprivation decile.
